# The “Dead Alive”

**Published:** 1859-08

**Authors:** R. B. Nason


### ECLECTIC DEPARTMENT,

### AND SPIRIT OF THE MEDICAL PERIODICAL PRESS.

---

### *The "Dead Alive."* By R. B. Nason, Esq., M. R. C. S.

[From the Montreal Medical Chronicle.]

Sir—An article, "The Dead Alive," in your January number, demands of me a veritable statement of the case alluded to. The subject of the inquiry is still living, and some time past has afforded me scope for observation.

I have only been waiting for a termination of the case, either in convalescence or death, to enable me to give the profession, through your valuable columns, a full and truthful history of this rare and curious case, replete with interest. The exaggerated statement which has gone the rounds of the press has produced such great curiosity in this immediate neighborhood, that I have been applied to by many parties, professional and non-professional, to be permitted to see the case, the parents of the patient having refused admittance to all strangers.

The case having extended over a long period, and fearing a detailed account might occupy too much of your valuable space, I have condensed the matter as much as possible; but should the profession consider the case worthy of a more enlarged history, I will gladly at some future period meet their wishes, as far as my rough notes, aided by my memory, will supply it.

In August, 1858, I was requested to visit Miss Amelia Hinks, aged twelve years and nine months, daughter of a harness-maker, and residing with her parents in Bridge street, Nuneaton. She was supposed to be suffering from pulmonary consumption. I found her much emaciated, and complaining of headache, great lassitude, loss of appetite, short cough, secretions morbid; catamenia not appearing. I prescribed an alterative to be taken at night, and a ferruginous tonic three times a day; a generous, though mild, nutritious diet, which she continued some time with benefit. I could not detect any chest disease. She then went into the neighborhood of Leanington, for change, to visit some friends, and after a short stay became much worse. Her parents, being apprised of her state, fetched her home as soon as possible. On her arrival I was requested to see her. I found her very attenuated, and complaining of great debility, headache, and loss of appetite; tongue clean; bowels confined. From this time she began to refuse food and medicine; and friends wished her not to be disturbed for anything, and daily and hourly anticipated her death. She was watched night after night, in anticipation of that event happening; and on the 18th of October, about half past 3 A.M, she apparently died. She is said to have groaned heavily, waved her hand (which was a promised sign for her mother to know that the hour for her departure was come), turned her head a little to the light, dropped her jaw, and *died.* In about half an hour after her supposed departure, she was washed, and attired in clean linen; the jaw was tied by a white kerchief; penny-pieces laid over the eyes; her hands, semi-clenched, placed by her side; and her feet tied together by a piece of tape. She was then carried into another room, laid on a sofa, and covered by a sheet; appeared stiff and cold; two large books were placed on her feet, and I have no doubt she was considered to be a sweet corpse.

About 9 A.M., the grandfather of the supposed dead, went into the death-chamber to give a last kiss to his grandchild, when he fancied he saw a convulsive movement of the eyelid, he having raised one of the coins. He communicated this fact to the parents and mourning friends, but they ridiculed the old man's statement, and said the movement of the eyelids was owing to the nerves working after death. Their theory, however, did not satisfy the experienced man of eighty years, and he could not reconcile himself to her death. As soon as I reached home, after having been out in the country all night, I was requested to see the child, to satisfy the old man that she was really dead. About half past ten A.M. I called; and immediately on my entrance into the chamber I perceived a tremulous condition of the eyelids, such as we frequently see in hysterical patients. The penny-pieces had been removed by the grandfather. I placed a stethoscope over the region of the heart, and found that organ performing its functions perfectly, and with tolerable force. I then felt for a radial pulse, which was easily detected, beating feebly, about 75 per minute. The legs and arms were stiff and cold; the capillary circulation gone. I carefully watched the chest, which heaved quickly, but almost imperceptibly; and immediately unbandaged the maiden, and informed her mourning parents that she was not dead. Imagine their consternation! The passing-bell had rung, the shutters were closed, the undertaker was on his way to measure her for her coffin, and other necessary preparations being made for interment. I ordered friction to the rigid limbs, moderately warm flannel to be applied, and other restoratives; and in about two hours she spoke, and requested to be taken to her mother's room, having been in the winding-sheet seven hours. She told her friends that she heard all they said, and knew they were laying her out; and that she heard the passing-bell ring, but could not speak. She passed a very large quantity of limpid urine, and refused food.

At 4 P.M. the following day she groaned heavily, bid the by-standers farewell, and relapsed into the same cataleptic state, and remained so six hours and fourteen minutes. I saw her in that state, and tried to raise her; she fell, listlessly, regardless of position or danger; and in whatever form the body was placed, it remained. She took no food between the attacks, but asked for water to wet her lips; and requested that nothing more in the shape of food might be given her, for she did not wish to eat or drink again until she did so in heaven. For a whole week she took nothing, but lay perfectly quiet, with her eyelids firmly closed, and her teeth in apposition. At the expiration of that time, I told the parents of the patient that I considered it their duty to insist upon food being taken. She was coaxed and threatened, but all in vain. She would not answer any question put to her, and whatever food was forcibly put into her mouth she ejected. I then, by means of a gag and an elastic tube, fed her with beef-tea, arrowroot, and other nutritious food. At this time she commenced moaning, and continued night and day, never ceasing for ten days.

After this painful state of things, her friends thought she must sink from exhaustion; but she did not seem to have sufficient power to stir; in whatever position she was placed, she remained, until changed by some attendants.

Her mother now drew my attention to the absence of kidney secretion, and assured me that for many days she had not voided urine. As there were more utensils in the room than the one set apart for her special use, I desired all to be removed but one, taking care that no other person made use of it. Ten days elapsed, but still no urine was discovered. I then told her mother that it was impossible—perfectly inconsistent with life; and asked if there were any closet or secret place in the room to which she had access. There was one, but it was filled with dirty linen. I asked permission to search it, when I found most of the linen saturated with urine. She had watched the opportunity of her friends' absence, and gone quietly into this closet and relieved her bladder.

At 2 A.M. one morning, whilst her parents were sleeping, she got out of bed, set fire to various articles in the room, and made her escape into the street in her night-dress, crying, "Murder!" The fire was, fortunately, extinguished, through the great presence of mind of the father, though at considerable cost, his hands being badly burned. She now began swearing most blasphemously, and continued to do so without intermission for sixty hours, after which she became exhausted, and relapsed into a state much resembling her former condition, in which state she has continued to the present time. Her eyelids, firmly closed, her teeth set fast, and muscles rigid. Her bowels are moved about once a week, and she passes urine daily into the bed; not, I believe, from any want of power of the sphincter. For the last month there has been great difficulty in feeding her by the mouth. The determination she evinced to resist food was extremely annoying; but I felt inclined to be as determined as she, and from that time have fed her three times a day by the rectum, giving her about half a pint of strong beef-tea and wine, alternately with the same quantity of new milk, arrowroot, eggs, &c. After the first lavements, and when I was prepared to operate the second time, she raised her hand to her mouth, and repeated the movement two or three times, evidently wishing to convey the impression that she preferred food to be administered in a more agreeable manner; but I found, on trying to give her food by the mouth, that she was obstinate as ever. I therefore persisted in administering food by the rectum. I ordered a certain number of biscuits to be placed on a chair near the head of the bed; the next morning they numbered one less. In the evening I requested the number might be made up; and three had vanished. We found unmistakable evidence that she had eaten them.

Dec. 4th.—Her friends beginning to despair of her, and feeling anxious to know what physical strength remained, as also whether she had the will to eat and power to masticate, I devised a scheme which, if carried out properly, would not only prove to her friends that she could open her eyes and mouth too if she thought she was unobserved, but in a great measure aid me in my diagnosis, and give me a hint as to my future treatment. I said in an audible tone, in the presence of the patient, that I insisted on the father and mother sleeping in another apartment; for she by her conduct was destroying their health. She should be locked in the room by herself all night. Having said so, we arranged that the father should be secreted in a closet in the chamber, with the door sufficiently open to allow him to watch her movements through the night. At 1 A.M., she raised herself upright in bed, opened her eyes, looked all around the room, turned down the bedclothes, and got out of bed as nimbly as ever, and walked directly to a quantity of food, which had purposely been laid for her. She turned it over, tasted, and finally took a good supply into bed, quietly drawing the bed-clothes over her.

8th.—It is five weeks to-day since she spoke to any one. Her eyelids have been closed the whole of the time, and her mouth, too, excepting when forcibly opened ; the pulse has varied very little, between 70 and 80. Her body appears much better nourished.

Having now given a description of the case to the present time, it remains for me to give my opinion as to the nature of this mysterious case. Considering that her mother has been at times hysterical, and that there has been sufficient evidence of the early development of the generative organs in most of the female members of the family, coupled with most of the prominent features of the case before us, and from many trifling, though important, incidents, which from defective memory I have omitted, I am inclined to consider it one of hysteria of an aggravated character, complicated probably with a morbid condition of the brain. I entertain hopes that, provided I can sufficiently nourish the body until the uterine organs are more fully developed, my patient may continue to be "*The Dead Alive!*"

I am, Sir, your obedient servant,
RICHARD BIRD NASON, M.R.C.S., L.S.A.
Bridge street, Nuneaton, *Dec.* 14, 1858.

